# Active vision in immersive, 360° real-world environments

**DOI:** 10.1038/s41598-020-71125-4

**Published:** 2020-08-31

**Authors:** Amanda J. Haskins, Jeff Mentch, Thomas L. Botch, Caroline E. Robertson

**Affiliations:** 1 Department of Psychological and Brain Sciences, Dartmouth College, Hanover, NH 03755 USA; 2 McGovern Institute for Brain Research, Massachusetts Institute of Technology, Cambridge, MA 02139 USA

**Keywords:** Human behaviour, Visual system, Psychology

## Abstract

How do we construct a sense of place in a real-world environment? Real-world environments are actively explored via saccades, head turns, and body movements. Yet, little is known about how humans process real-world scene information during active viewing conditions. Here, we exploited recent developments in virtual reality (VR) and in-headset eye-tracking to test the impact of active vs. passive viewing conditions on gaze behavior while participants explored novel, real-world, 360° scenes. In one condition, participants actively explored 360° photospheres from a first-person perspective via self-directed motion (saccades and head turns). In another condition, photospheres were passively displayed to participants while they were head-restricted. We found that, relative to passive viewers, active viewers displayed increased attention to semantically meaningful scene regions, suggesting more exploratory, information-seeking gaze behavior. We also observed signatures of exploratory behavior in eye movements, such as quicker, more entropic fixations during active as compared with passive viewing conditions. These results show that active viewing influences every aspect of gaze behavior, from the way we move our eyes to what we choose to attend to. Moreover, these results offer key benchmark measurements of gaze behavior in 360°, naturalistic environments.

## Introduction

Constructing a sense of place in a complex, dynamic environment is an active process. Humans actively sample their sensory environment to build an understanding of their surroundings and gain information relevant to their behavioral goals^1,2^. Yet, much of what we know about how people encode real-world environments comes from paradigms that severely limit participants’ active affordances: paradigms in which head-restricted participants view images that are passively displayed on a computer screen. In this context, the participant’s behavioral repertoire is limited to eye movements, and the displayed portion of an environment is typically limited to a single field of view. In contrast, everyday visual environments are actively explored. We gain rich information about a place by shifting our eyes, turning our heads, and moving our bodies. This is because real-world scenes are immersive, extending 360° around us and beyond any single field of view. How does scene understanding unfold in immersive, active viewing conditions?

It has long been understood that active viewing conditions impact perceptual processing. Neurons in early stages of the visual system are sensitive to the distinction between self- and world-generated motion under conditions that are carefully matched for equivalent retinal stimulation and attentional engagement^3,4^. Further, active vision is thought to be necessary for typical visual development: even basic visual functions, such as depth perception and contrast sensitivity, suffer when animals are denied self-motion in a visual environment, but are passively exposed to equivalent visual stimuli^5^. Studies in humans also suggest that perceptual systems differentially represent stimuli that are encountered via active vs. passive viewing^1^. For example, self-motion and vestibular cues are critical for accurately inferring an object’s distance^6,7^ and three-dimensional structure^8^; moreover, an object’s spatial location is better recalled when it has been actively reached for rather than passively moved toward^9^. Until recently, a lack of available technologies has limited experimenters’ ability to study active vision under naturalistic conditions^10^, and in particular, no studies have explored how active viewing conditions impact the way that humans process complex visual stimuli, such as real-world scenes.

Here, we used a novel experimental design to study real-world scene processing during active and passive viewing conditions. We exploited recent developments in virtual reality (VR) to immerse participants in real-world, 360° scenes. Meanwhile, we monitored participants’ gaze using in-headset eye-tracking as they explored these environments, revealing which scene regions they prioritized over others as they built an understanding of the scene. In one condition, participants explored scenes from an active, first-person perspective. In the other condition, scenes were passively displayed to participants while they were head-restricted in a chin rest. In both conditions, diverse, real-world scenes were displayed with the same wide-angle field of view (100 DVA), and participants were exposed to comparable portions of the display over the course of the trial. Thus, this paradigm enabled us to perform quantitative, in-depth comparisons of gaze behavior and attentional deployment as subjects encoded a diverse set of novel, real-world scenes during active vs. passive exploration.

Our central hypothesis was that active viewing conditions would increase a viewer’s exploratory, information-seeking behavior in a real-world scene. We tested this by measuring the degree to which participants’ overt attention was dominantly predicted by the spatial distribution of scene features that are semantically informative (e.g., objects, faces, doors)^11,12^, as compared with scene regions that are rich in salient visual features (e.g., luminance, contrast, color, and orientation)^13,14^. Previous studies have shown that these information sources compete for participants' top-down vs. bottom-up attention as scene understanding unfolds, although attention is predominantly predicted by the distribution of semantic information^12,15^.

In brief, we observed that participants’ attention was dominantly guided by semantic meaning as compared to low-level visual features in both active and passive conditions, replicating previous findings^12,15^. Crucially, this effect tripled during active viewing, reflecting an increase in signatures of top-down attentional guidance when participants were free to actively view their environment. Moreover, in service of this information-seeking behavior, active viewers made shorter, more exploratory fixations as compared to passive viewers. These results have broad implications for studies of visual cognition, suggesting that active viewing influences every aspect of gaze behavior—from the way we move our eyes to what we choose to attend to—as we construct a sense of place in a real-world environment.

## Results

**Figure 1:**
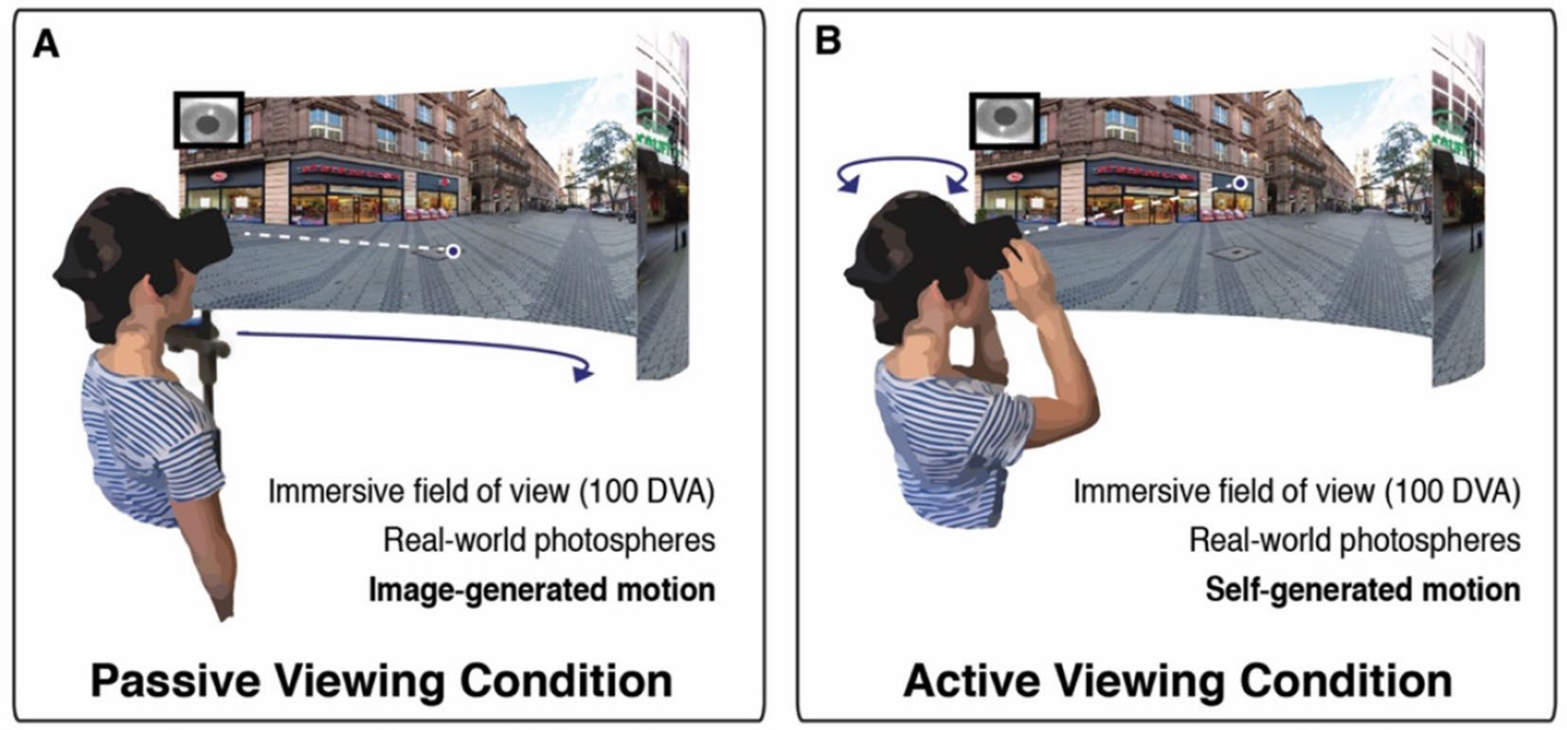
Passive viewing condition vs. active viewing condition. On each trial, participants viewed immersive, 360° real-world scenes via a headmounted VR display while gaze position was monitored using an in-headset eye-tracker. (**A**) In the passive condition, participants were head-restricted using a chin rest, and scenes panned across the display. (**B**) In the active condition, participants explored scenes from a first-person perspective through movement of their eyes, head, and body.

**Figure 2:**
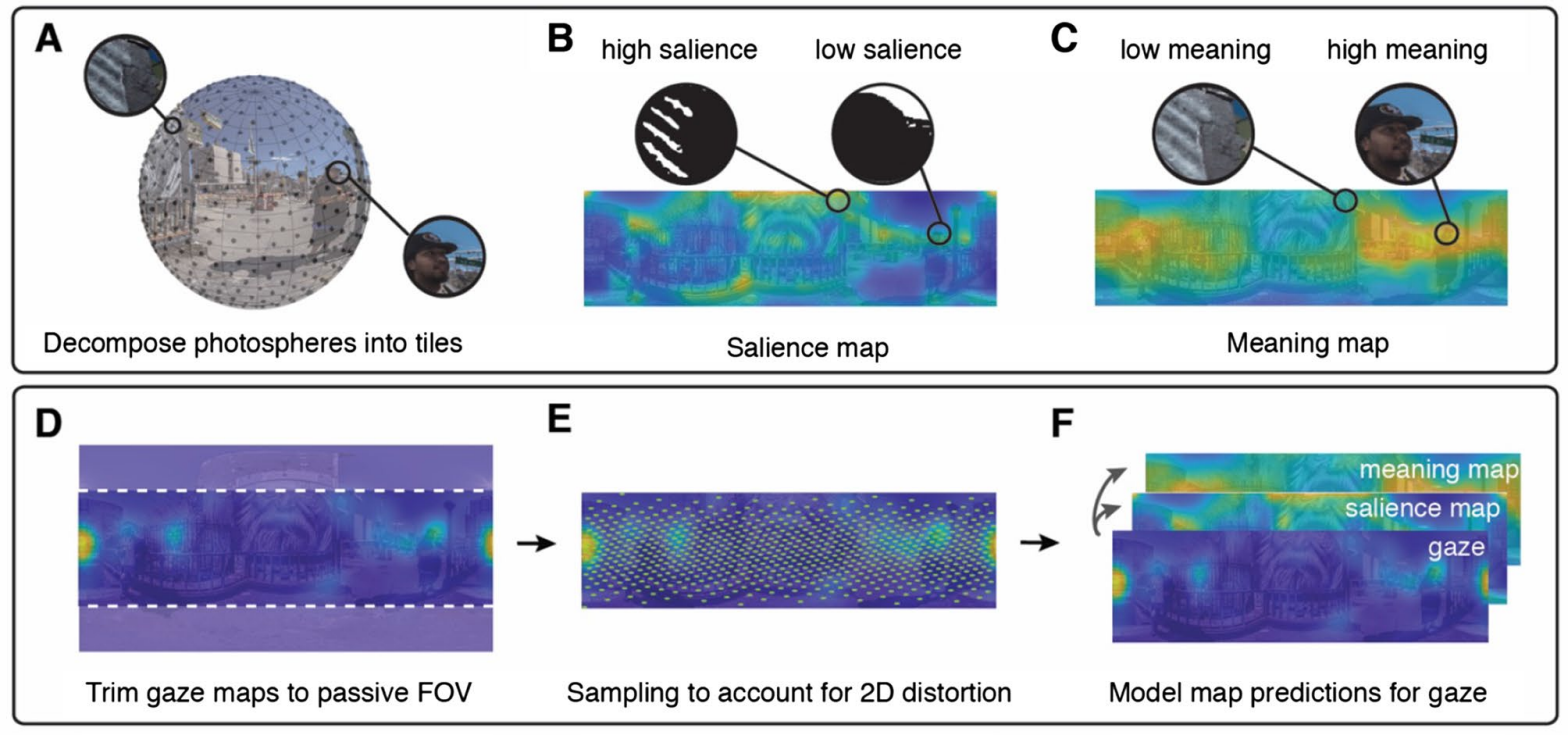
Comparing salience and meaning maps to gaze behavior. (**A**) Each photosphere was first decomposed into smaller undistorted image tiles. Next, we created two models of the content in each real-world environment. (**B**) “Salience maps” were generated by modeling low-level visual features for each tile using the GBVS Toolbox^16^. Each tile was then projected onto a two-dimensional salience map. (**C**) “Meaning maps” were generated via online participants who rated the semantic content, or “meaning” of each image tile. Each tile’s rating was then projected onto a two-dimensional meaning map. (**D**) Group gaze maps were trimmed (vertically) to match the passive condition field of view. (**E**) Points are sampled evenly on a sphere and used to account for photosphere distortion in two-dimensional maps. (**F**) A linear mixed effects model was used to compare the degree to which each model predicted attentional guidance in our two conditions. (Image adapted from Pyramid Oracle Panorama by *Nathan Tweti*.)

To test whether active viewing conditions modulate attentional guidance in real-world scenes, we designed a novel eye-tracking paradigm to directly compare eye movements while participants viewed immersive, 360° environments in two experimental conditions. During the passive condition, participants’ heads were fixed in a chin rest, and the scene panned across the HMD screen (Fig. 1A). In the active condition, participants could actively explore the environment via self-directed motion (i.e., saccades, head, and body turns) (Fig. 1B). With the exception of fixation number and duration, all analyses we report here compare viewers’ gaze behavior only within the regions visible to passive viewers (Fig. 2D). We observed high-quality eye-tracking in the HMD, comparable with that reported in fixed display studies at screen center (ideal observer accuracy: 0.79 DVA, precision, 0.11 DVA; see Supplemental Fig.1). In addition, accuracy following calibration did not differ between participants in the active (*M* = 2.69 ± 0.38 DVA) and passive (*M* = 3.29 ± 0.40 DVA) conditions (*t*(15) = -1.10, *p* = 0.29).

Our central hypothesis was that active viewing conditions would increase exploratory, information-seeking behavior in a real-world scene. To test this hypothesis, we first constructed two models of the visual content in each environment (Fig. 2, “Methods”). First, we computed a traditional "salience map”, which reflects the distribution of low-level visual features in a scene (e.g., contrast, color, orientation, etc.)^16^. Prominent low-level visual features in a scene are known to predict a significant portion of gaze behavior^13,17^. However, as attention is drawn on the basis of visual salience, rather than semantically meaningful scene regions, the degree to which such maps predict gaze behavior is not related to information-seeking behavior, thus providing a good baseline model for our hypothesis.

Second, we computed a “meaning map” for each scene, which reflects the distribution of high-level semantic features in an environment (e.g., faces, objects, doors, etc.)^12^. “Meaning maps”, a recently proposed “conceptual analogue” of salience maps^12^, reflect the spatial distribution of features that are relevant to understanding the semantic content and affordances available to the viewer in a scene. Recent studies have shown that attentional deployment in novel scene images is dominantly predicted by the spatial distribution of scene features that are semantically informative (meaning maps) as compared with low-level features (salience maps)^12,15^. Consistent with cognitive guidance theories of attention^18,19^, these studies emphasize the notion that overt attention primarily reflects information-seeking behavior on the part of a viewer.

We specifically hypothesized that active viewing conditions would increase exploratory, information-seeking behavior, and therefore increase the degree to which meaning-maps predict gaze behavior. One of the few existing observations from eye-tracking in VR is the tendency for viewers to fixate near the equator^20^; therefore to test our hypothesis, we first generated a map of the equator to serve as a baseline prediction for 360° viewing behavior. Then, for each condition, we compared the additional predictive contribution of each map of environmental content (salience map, meaning map) using a linear mixed-effects model. Specifically, we included three spatial maps (i.e., salience maps, meaning maps, and our baseline map of the equator) and viewing condition as fixed effects and individual scenes as random effects in the model. All results are summarized in Table 1.

**Table 1:** Linear mixed effects model results summary. Main effects, two-way interactions, and three-way interaction of fixed effects included in linear mixed regression using the model formula: *gaze* ~ condition*meaning*salience + *condition:equator* + *(1 | scene).* We observed significant main effects of condition, meaning, and salience on gaze, significant condition:meaning and condition:salience interactions, and critically, a significant three-way interaction between condition, salience, and meaning.

	numDF	denDF	*F* value	*p* value
Condition	1	6,163,960	8,818.25	< 0.001
Meaning	1	6,163,907	71,586.82	< 0.001
Salience	1	6,163,982	19,920.19	< 0.001
Condition:meaning	1	6,163,960	21,579.7	< 0.001
Condition:salience	1	6,163,960	212.22	< 0.001
MEANING:salience	1	6,163,997	64,200.96	< 0.001
Condition:equator	2	6,163,980	485,605.49	< 0.001
Condition:meaning:salience	1	6,163,960	71.36	< 0.001

Overall, we observed that overt attention in real-world scenes primarily reflects information-seeking behavior in both active and passive conditions, confirming previous results^12,15^. Both salience and meaning maps significantly predicted participants’ overt attention (salience estimated marginal effect: 0.15 ± 0.03 STE, CI [0.09,0.21]; *p* < 0.001; meaning estimated marginal effect: 0.31 ± 0.03 STE, CI [0.25, 0.36]; *p* < 0.001). However, in both active and passive conditions, meaning was significantly more predictive of which scene regions participants explored than salience (meaning:salience interaction: *p* < 0.001; post-hoc corrected t-tests for meaning vs. salience: *p* < 0.001 for both conditions).

**Figure 3:**
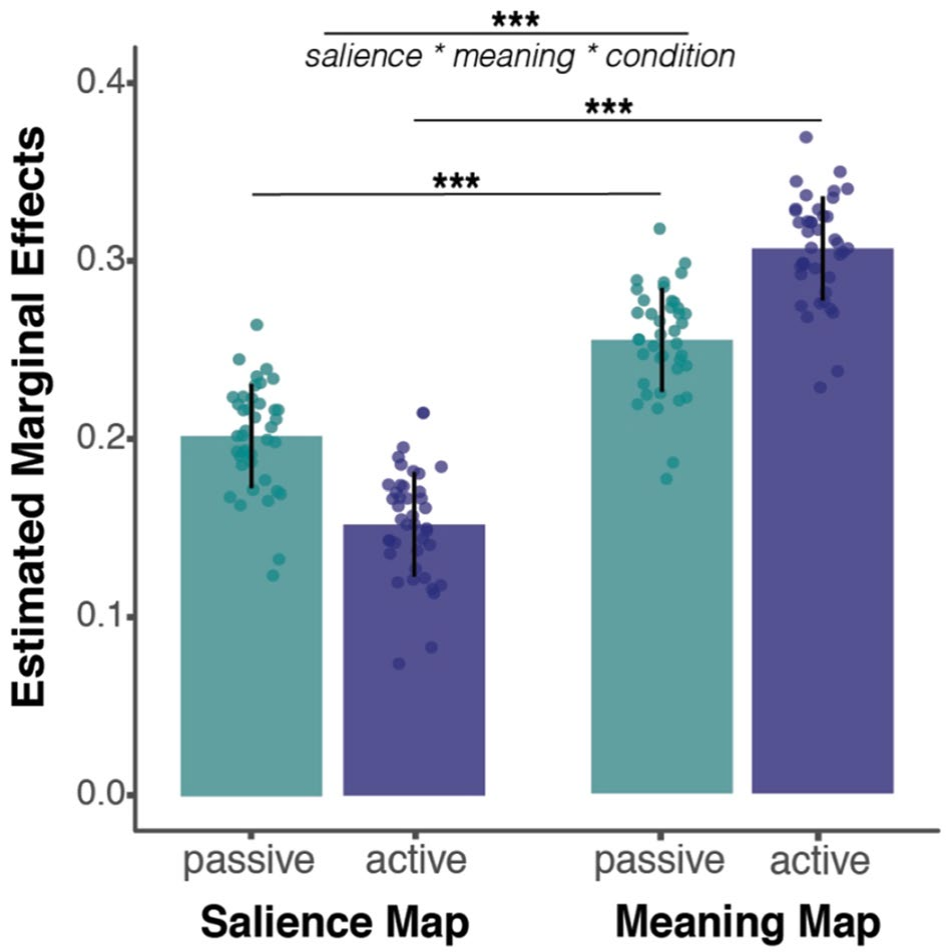
Active viewing increases top-down attentional allocation. Estimated marginal effects of salience and meaning maps on predicting overt attention in each condition (active vs. passive). We found that viewing condition (active vs. passive) significantly modulated gaze behavior (salience*meaning*condition: *p* < 0.001). Specifically, active viewers disproportionately directed their attention to meaningful, over salient, scene regions. Individual points represent random item effects (i.e., individual scenes). Error bars represent prediction intervals (± 1 STE). *** denotes *p* < 0.001.

Critically, however, this advantage for meaningful scene regions nearly tripled in the active as compared with the passive condition (salience*meaning*condition: *p* < 0.001; Fig. 3). Post-hoc analyses revealed that the estimated marginal effect of meaning (i.e., its predictive contribution, holding other factors constant) was significantly greater for active as compared with passive viewers (passive: 0.25 ± 0.03 STE; active: 0.31 ± 0.03 STE; *p* < 0.001). Conversely, the estimated marginal effect of salience was greater in the passive condition than in the active condition (passive estimate: 0.20 ± 0.03 STE; active estimate: 0.15 ± 0.03 STE; *p* < 0.001). Taken together, these results show that active viewing conditions specifically increase attentional allocation to semantically relevant regions of a visual environment.

Control analyses confirmed that these results could be attributed to active affordances rather than passive viewers’ limitations. First, we restricted our analysis to the fields of view containing regions ranked in the top 50^th^ percentile for meaning. The disproportionate advantage for meaning-guided attention in the active condition remained significant even after eliminating less meaningful scene regions that passive viewers were required to scan as a result of the panning image presentation (Supplemental Table 1). Second, due to the panning image presentation, participants in the passive condition were only able to revisit scene regions for a portion of the trial (i.e., approximately 5 s), whereas active participants could revisit scene regions at any point during the 20-s trial. Thus, we reasoned that differences in viewers’ attention could have been the result of different sampling (and re-sampling) opportunities. To rule out this account, we conducted a second control analysis in which we capped active viewers’ maximum viewing times for any scene region. To do this, we first identified groups of fixations made by a subject within 90° of one another. We then downscaled any group that exceeded a total duration of 5 s, thus equating the maximum time that active and passive viewers could have explored any portion of a scene. The downscaling procedure impacted less than 3 percent of active fixations. When gaze maps were computed using downscaled fixations, the advantage for meaning-guided attention among active viewers remained significant (Supplemental Table 2). Thus, as predicted, in the active condition, when participants were free to move their head and body, participants’ attention was disproportionately directed towards semantically relevant regions of a visual environment.

**Figure 4:**
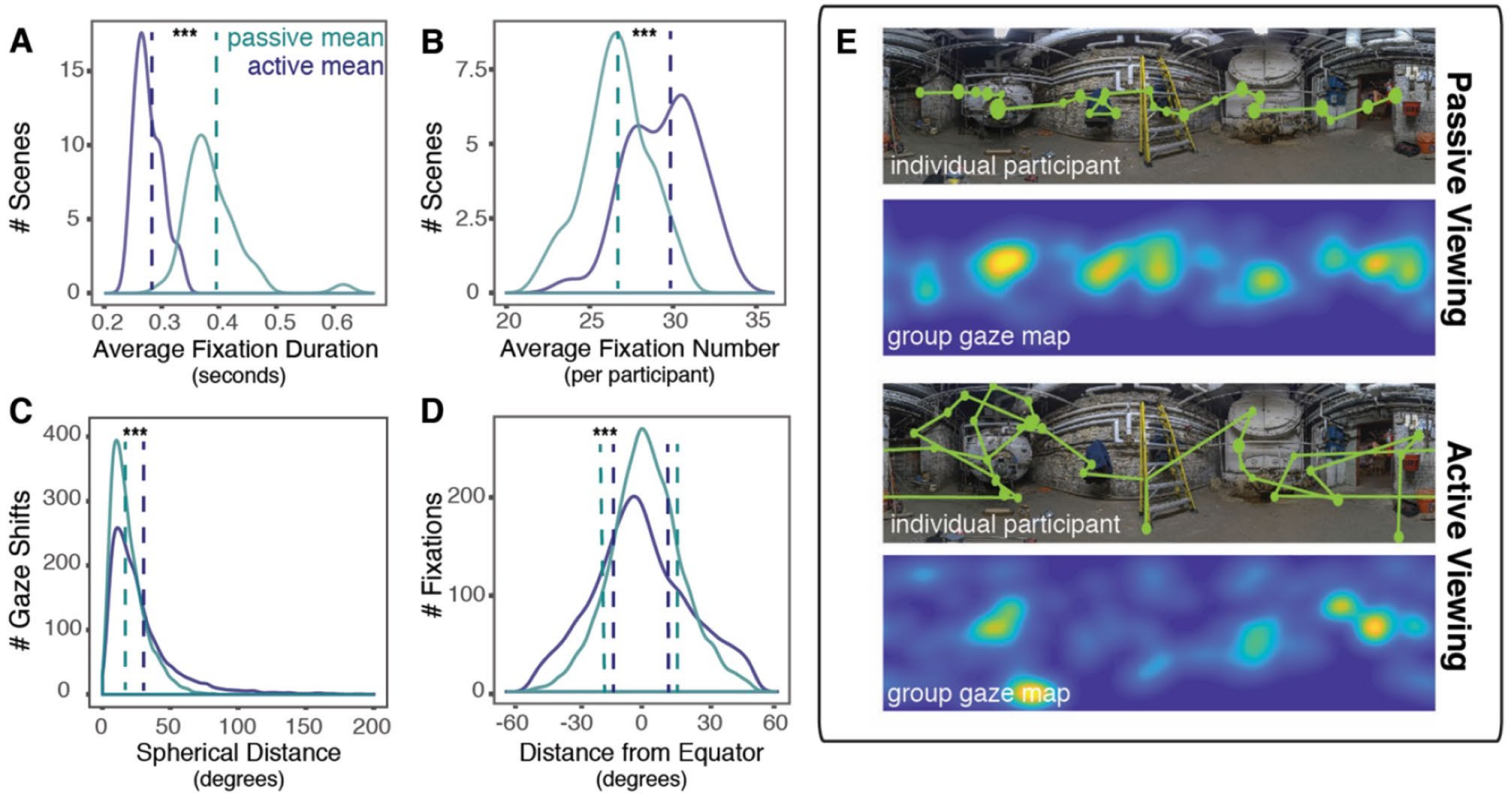
Active viewing impacts eye movements. (**A**) Relative to fixations made in the passive viewing condition, fixations in the active viewing condition were shorter (**B**) and more frequent. (**C**) Gaze shifts in the active viewing condition were also larger, and (**D**) the spatial distribution of gaze in the active viewing condition was less centrally tending. (**E**) Sample duration-weighted fixations and gaze shifts made by a single participant (top) and group fixation map for participants (bottom) per condition. ***Denotes *p* < 0.001. *(Image * *adapted from* Old&New Boiler Panorama *by Nathan Tweti)*.

We further characterized gaze behavior during active viewing using two measures of spatial distribution that are independent of individual scene content: 1) deviation from center bias and 2) entropy. Prior to the observation of an equator bias in 360° environments^20^, studies of visual attention using traditional fixed displays also commonly observed a bias to fixate near the center of an image^21,22^. Although the precise source of this bias is disputed^17,23,24^, deviation from “center bias” has been used to describe the degree of visual exploration^25^. On average, we found that fixations made in the active condition had less center bias, or were further from the equator^20^, (17.13° ± 0.54 STE) than fixations made in the passive condition (12.53° ± 0.23 STE) (*t*(39) = 9.98, *p* < 0.001; Fig. 4). Of course, this result could reflect a systematic, non-central bias (e.g., active viewers could have routinely looked toward the poles), rather than more exploratory gaze behavior per se. To address that possibility, we used a second measure, entropy, a measure of homogeneity (or lack thereof) in the probability distribution of fixations^26^, to test whether any systematic biases occurred in each viewing condition. We found that gaze behavior in the active condition was more entropic (3.49 ± 0.07 STE) than gaze in the passive condition (2.88 ± 0.02 STE) (*t*(39) = 8.63, *p* < 0.001). Taken together, these results further demonstrate that active viewers prioritize rapid exploration of new scene regions that are rich in semantic content, relative to passive viewers.

Finally, we sought to characterize the eye movements participants made in service of this increase in information-seeking behavior (Fig. 4). Participants made shorter (0.27 s ± 0.004 STE) and more frequent (29.45 ± 0.34 STE) fixations in the active as compared with the passive condition (size: 0.39 s ± 0.008 STE, *t*(39) = − 14.52, *p* < 0.001; frequency: 26.60 ± 0.31 STE, *t*(39) = 7.53, *p* < 0.001. Further, active viewing conditions impacted the magnitude of gaze shifts (i.e., the distance between two fixations) participants made while exploring their environment. Gaze shifts were significantly larger in the active condition (29.17 DVA ± 0.48 STE) as compared with saccades made in the passive condition (19.08 DVA ± 0.23 STE: *t*(39) = 23.11, *p* < 0.001). Indeed, it was not uncommon for active viewers to make gaze shifts as large as 150 DVA, exceeding previous saccade length estimates from fixed display studies (5–10 DVA) by an order of magnitude^27,28^. Of course, gaze shifts of this size are only possible because active viewers could move both their eyes and head. Notably, our headmounted display subtended a wider field of view (100 DVA) than typically afforded by fixed display studies. Importantly, this wide display was matched for both active and passive conditions; thus, the differences we observed cannot be attributed to the size or content of our stimuli, which is known to impact the size of gaze shifts^29^. Instead, our results demonstrate that active viewing conditions fundamentally impact the size of gaze shifts a viewer will routinely choose to make when exploring a real-world visual environment: people make faster fixations and larger gaze shifts, once more suggesting more exploratory gaze behavior.

## Discussion

Our results provide novel insights into the process by which we actively construct representations of immersive, real-world visual environments. We found that, when participants are unconstrainted and free to choose their field of view, their behavior is most guided by meaningful, semantic properties of the environment, as compared with passive viewers. Moreover, in service of this information-seeking behavior, active viewers employ shorter, more entropic fixations. All in all, we demonstrate that active viewing conditions impact nearly all features of gaze behavior, from gaze mechanics (how we move our eyes) to gaze dynamics (what we choose to attend to). These findings lay the foundation for future studies of gaze behavior in immersive, real-world environments using VR.

The recent development of eye-tracking in immersive VR is a critical advance for investigating real-world scene processing in the context of natural behavioral affordances (see Scarfe and Glennerster^10^, for review). VR frees the subject from the limitations typically imposed by head-restricted display studies, allowing for eye, head, and body movements^1^. Moreover, it does so without sacrificing experimental control. Active participants in our study were not only free to naturally explore an environment from a first-person perspective; they were also shown a large, diverse set of stimuli within precise trial structures in a counterbalanced design. Such stimulus diversity and controlled presentation is an essential ingredient for quantitative, model-based insights into active human gaze behavior. This opportunity for experimental control and diverse stimulus presentation stands in contrast to mobile eye-tracking paradigms, where participants might only traverse a single, extended environment (e.g., a university campus) in which the low-level visual features (e.g., the lighting/contrast) as well as high-level visual features (e.g., people walking down the street) inevitably vary between participants. Previous approaches comparing active versus passive viewing have relied on a combination of fixed-display and mobile paradigms^30,31^, which inherently differ in terms of task demands (e.g., watching a video vs. navigating) and displayed field of view. All in all, eye-tracking in immersive VR is an exciting opportunity for novel insight into active human gaze behavior.

Our findings have multiple implications for active, real-world vision. Recent studies have proposed a dominant role for semantically meaningful scene regions in guiding attention even during passive viewing of fixed-display images, suggesting that gaze behavior primarily reflects high-level information-seeking priority as scene understanding unfolds^11,12,15^. Our results extend these findings in three key ways. First, we demonstrate that semantically relevant features guide attention in immersive, naturalistic environments. This is an important demonstration if semantically guided attention is indeed a feature of real-world vision. Second, we show that active viewing conditions increase the advantage for semantics over low-level visual salience in guiding gaze behavior. Again, this finding has important implications for real-world vision; our results suggest that when participants are free to seek out information and choose their field of view, their behavior is most guided by meaningful, semantic properties of the stimulus, or scene. Finally, our findings provide key benchmark measurements of gaze behavior in diverse, real-world scenes during active viewing conditions, demonstrating that active viewers make quick, entropic fixations and shift their gaze nearly twice per second.

We attribute the observed increase in exploratory, information-seeking behavior in active viewing conditions to differences in the *affordances* available to active viewers, not differences in their *action goals*. Our experiment manipulated self- vs. image-generated motion, but not participants’ task, a factor long understood to impact gaze behavior^32–34^. Participants in the active condition had no objective to physically interact with meaningful scene regions, such as objects; nor was this a possibility. Yet, access to a naturalistic behavioral repertoire—a broader capacity for self-generated action—nonetheless impacted how participants moved their eyes and deployed their attention. Our results are consistent with theoretical models linking perceptual processing and motor plans in a perception–action cycle^35^, in which perceptual processes depend on movement states^36–39^ and the role of vision is to provide evidence to satisfy behavioral goals^1^. These findings have important implications for future work investigating cognitive processes where motor goals are often hampered by less naturalistic paradigms, such as scene perception, spatial memory, and even social inference.

Of course, our experimental approach also has drawbacks. The design of our passive condition was limited by the known tendency for image-generated motion to induce participant motion sickness^40^. As a result, we opted to implement a passive condition that slowly revealed the panoramic environments to participants, giving passive viewers the opportunity to explore the same fields of view as active viewers, but did not provide an exact match for the sequence of moment-to-moment fields of view that an active viewer would have taken in a scene. We do not think that these limitations impacted our results. As both semantic and salient scene features were equally distributed around our panoramic scenes, participants in both conditions were given an equal amount of time to explore each environment, and our control analyses demonstrate that active viewers rarely, if ever, dwelled on any portion of the panoramic scene for longer than it would have been displayed to a passive viewer. Thus, the disproportionate advantage for meaning over salience in predicting gaze behavior during active viewing conditions can be attributed to which scene features a participant chose to attend to in any field of view, rather than which fields of view active vs. passive viewers sampled during a trial. In our active condition, we were limited by the challenge of self-embodiment^40^: participants could move their eyes, head, and body, but their movements were not paired with the typical visual experience of seeing one’s body. It is possible that our results would strengthen if the perception of self-embodiment were afforded to active viewers, as in real-world viewing conditions. Finally, given the free viewing nature of our experiment, we cannot rule out the possibility that our effects are mediated by differences in attentional engagement between our two conditions, particularly given that VR was a novel experience for most participants. To address this possibility, future investigations may seek to increase participants’ agency and potential for engagement; for example, an intermediary viewing condition might allow participants situated in a chin rest to select their field of view via joystick, as compared to head movement.

In sum, our results demonstrate for the first time that active viewing conditions impact attentional deployment in 360°, real-world scenes. Signatures of top-down attentional guidance increase in active viewing conditions: active viewers disproportionately allocate their attention to semantically relevant scene features using quicker, more exploratory eye-movements than passive viewers. In addition to providing key benchmarks for active, naturalistic gaze behavior, these results show that active viewing influences every aspect of gaze behavior, from the way we move our eyes to what we choose to attend to.

## Methods

### Participants

Eighteen adults participated in the main experiment (thirteen females; mean age 22 ± 3.73 STD years). Three additional participants completed a pilot study designed to test eye-tracker accuracy and precision (Supplemental Fig. 1). Participants were recruited based on (1) having normal or contact-corrected vision and no colorblindness, (2) having no neurological or psychiatric conditions, and (3) having no history of epilepsy. All participants gave written informed consent. The experiment was performed in accordance with relevant guidelines and regulations, and all experimental procedures were approved by the Dartmouth College Committee for the Protection of Human Subjects (CPHS) and Massachusetts Institute of Technology Committee on the Use of Humans as Experimental Subjects (COUHES).

### Stimulus and headmounted display

Stimuli consisted of 360° “photospheres” of real-world scenes, sourced from an online photo sharing website (www.flickr.com). Photospheres depicted a diverse set of indoor and outdoor settings with content including people and objects. Each photosphere was applied to a virtual environment built in Unity version 2017.3.1f1 (www.unity3d.com) and integrated with a headmounted display (Oculus Rift, Development Kit 2, www.oculus.com, low persistence OLED screen, 960 × 1,080 resolution per eye; ~ 100° field of view; 75 Hz refresh rate).

### Eye-tracker specifications and accuracy

A monocular, in-headset eye-tracker (Pupil Labs: 120 Hz sampling frequency, 5.7 ms camera latency, 3.0 ms processing latency; 0.6 visual degrees accuracy, 0.08 visual degrees precision) continuously monitored the position of participants’ right eye during scene viewing. Eye movements were recorded using custom scripts written in C# for Unity. Refer to Supplemental Fig. 1 for observed accuracy and precision of Pupil Labs eye-tracker at varying distances from screen center.

### Experimental procedures

On each trial of the experiment (40 trials), participants were presented with a photosphere via the headmounted display (HMD). Participants were instructed to “fully and naturally explore each scene”. Participants were given a break after every 10 scenes, after which the eye-tracker was recalibrated.

There were two viewing conditions in this experiment: active and passive (Fig. 1). In both conditions, the stimulus was presented via the HMD and each trial lasted for 20 s. During the active condition, participants stood while wearing the HMD and actively explored the photosphere via self-directed eye movements and head turns. In contrast, during the passive condition, participants’ heads were fixed in a chin rest and the scene panned across the screen, rotating 360° at a constant velocity (22°/ second). In order to prevent the sensation of motion sickness, rotational velocity gradually ramped up and down during the first and last two seconds of each passive trial.

There were 40 total stimuli included in the experiment. Each participant viewed 20 stimuli in each of the two conditions with condition assignment randomized for each trial and participant. Conditions were blocked, but condition order was counterbalanced across participants. The initial rotation angle of each scene was held constant across participants.

### Practice trials and calibration routine

There were three phases to the experiment: practice, calibration, and experimental trials. During the practice phase, participants performed two active condition trials. This ensured that participants had acclimated to the virtual environments prior to starting the experiment. Following the practice phase, participants performed a 14-point calibration routine in order to validate eye-tracking accuracy. Participants repeated the calibration routine after every 10 experimental trials. A confidence threshold of 0.8, the default pupil detection threshold specified by Pupil Capture eye-tracking software, was used to validate calibration routines. Failed calibration routines were repeated before proceeding to experimental trials.

After each trial in the Experimental Phase, participants returned to a virtually rendered home screen where they were instructed to take a break. Upon advancing from the home screen, participants were presented with a pre-trial fixation screen with a target at screen center. Participants were instructed to fixate on the target so that gaze drift could be assessed. If significant drift (> 5° visual angle) was detected, a recalibration routine was performed.

### Eye-tracking data analysis

Raw *x (*and *y)* gaze points were converted from normalized screen coordinates to DVA using the following equation^41^:

$$B_{a} = \left( {\frac{{180^{ \circ } }}{\pi }} \right) \cdot \left( {\frac{{2 \cdot FOV_{a} }}{{FOV_{\max } }}} \right) \cdot atan2\left( {p_{a} ,d} \right)$$

where, *B*_*a*_ denotes the azimuth (or elevation) angle of gaze position in visual degrees relative to screen center, *FOV*_*a*_ denotes the field of view of the x (or y) dimension of the HMD as a proportion of the largest dimension of the HMD (i.e., 100 DVA), *p*_*a*_ denotes the gaze position in normalized screen coordinates, and *d* denotes the distance in Unity units that places the participant at the origin of the spherical eye-tracking coordinate system.

Next, gaze coordinates were rectified with head position (pitch, yaw, roll), and transformed into latitude and longitude positions on a sphere (spherical degrees):

$$\begin{aligned} Lat & = x - \left( {B_{y} \cos (z) + B_{x} \sin (z)} \right) \\ Long & = y + \left( {\cos (Lat) \cdot \left( {B_{y} \cos (z) + B_{x} \sin (z)} \right)} \right) \\ \end{aligned}$$

Here, *x* denotes pitch in spherical degrees (with up being negative and down being positive), *y* denotes yaw in spherical degrees, and *z* denotes roll in radians. *B*_*x*_ and *B*_*y*_ denote gaze point distance from screen center in visual degrees.

Within each trial, a gaze point was labelled as invalid if: (1) it fell outside the field of view (i.e., greater than 50° from screen center in either the *x* and/or *y* direction), (2) pupil detection confidence was low (i.e., below 50 percent), or (3) no data was collected (e.g., during a blink). Trials with more than 75 percent of points labelled as invalid were excluded from the analysis.

### Defining fixations

Next, to determine fixations, the orthodromic distance and velocity was calculated between consecutive gaze points. Specifically, the mean absolute deviation (MAD)^42^ in gaze position was calculated within a seven-sample sliding window (~ 80 ms) and potential fixations were defined as windows with a MAD less than 50°/s^31^. Potential fixations were concatenated if two group centroids were displaced by less than 1° and the two potential fixations occurred within 150 ms of each other. Fixations with durations shorter than 100 ms were excluded^31,43^.

### Quantifying central tendency

A routine observation in fixed display studies is the tendency for fixations to be disproportionately allocated at the center of a scene^22^. “Central tendency” has been employed as a metric of visual exploration, where fixations that are less centrally tending are considered more exploratory^25^. Given the tendency for viewers to fixate near the equator in VR^20^, “equator bias” was calculated per condition by averaging the distance of each fixation from the equator in the *y* dimension only. Fixations made in the active condition were excluded if they fell beyond the region displayed during the passive condition.

### Fixation density map generation

We next characterized the spatial distribution of fixations on each trial. To generate two-dimensional fixation density maps, we first plotted the fixations for all subjects in a given scene and condition in equirectangular space. To standardize fixation density maps across conditions, fixations in the active condition were excluded if they fell beyond the region displayed during the passive condition (27.9 percent of the spherical scene; Fig. 2D). Given the infrequency with which participants looked at the upper and lower poles of a scene, only 12.2 percent ± 1.29 STE of fixations were discarded from the active condition. Additionally, because the rotational velocity was changing at the beginning and end of each passive condition trial, fixations made during the first and last two seconds were excluded from both active and passive conditions. The resulting fixation maps were smoothed with a variable-width gaussian filter (“modified gaussian”^44^) to account for distortions of the equirectangular image at shorter latitudes (i.e., approaching the poles). Specifically, the width of the gaussian filter is scaled by the latitude of the gaze point using the following equation:

$$a = \frac{{B_{w} }}{\cos (Lat)}$$

where the width of the filter, *a*, at a given latitude (*Lat*) has been scaled from the base filter width (*B*_*w*_) applied at the equator.

### Quantifying entropy

The entropy of fixation density maps (described above) was calculated using the following equation^26^:

$$E = - \sum {p \cdot \log_{2} p}$$

where p contains the fixation density map’s histogram counts.

Because entropy estimates can be impacted by small sample sizes^45^, and because an uneven number of fixations were made across conditions for any single scene, we applied a bootstrapping technique to estimate entropy. Across 100 iterations, we randomly sampled 24 fixations from each participant within each scene in a given condition. The target of 24 fixations was chosen in proportion to previous studies analyzing the entropy of nine fixations per 6 s trial^25,46^.

### Gaze map generation

Finally, we generated group gaze maps by plotting duration-weighted fixations made by all subjects per scene and condition (Supplemental Fig. 2). In order to prevent extreme fixation durations made by individual subjects from exerting an outsized impact on the group gaze maps, fixations with durations above the 95^th^ percentile were reduced to the 95^th^ percentile value^12,15^. Additionally, each individual subject’s fixations were normalized on a scale from 0.1 to 1.

### Salience map and meaning map generation

To assess the impact of active viewing on the spatial distribution of gaze, we generated two continuous content maps for each scene: (1) a “salience map”, a map of low-level visual salience and (2) a “meaning map”, a map of high-level semantic features (i.e., “meaning”). Recent approaches using continuous meaning maps to quantify semantic-level salience in a scene have enabled the direct comparison of bottom-up vs. top-down contributions to attentional guidance^12^.

Salience maps were generated using the Graph-Based Visual Saliency (GBVS) Toolbox^16^. Each photosphere was uniformly sampled and decomposed into a set of 500 square tiles, each with a diameter of 7.5°. The GBVS model with default feature channels (i.e., color, intensity, orientation) was applied to each tile, which was then projected back to its position in the equirectangular image. Salience maps were smoothed using the variable-width gaussian filter applied to gaze maps.

To generate meaning maps, we applied the procedures described by Henderson and Hayes^12,15^ to 360° scenes. Each photosphere was uniformly sampled at both coarse (100 points) or fine (500 points) spatial scales and decomposed into sets of partially overlapping circular tiles with diameters of 20.6 spherical degrees or 7.5 spherical degrees, respectively. Scene tiles were produced by generating a rectilinear projection (1,100 × 1,100 pixels) around each point sampled on the sphere. Each coarse tile was down-sampled to match the resolution of fine tiles, resulting in a diameter of 150 pixels for all scene tiles. The full scene tile stimulus set contained 20,000 unique fine-scale tiles and 4,000 unique coarse-scale tiles, for a total of 24,000 scene tiles.

A total of 1,879 participants on Amazon Mechanical Turk rated scene tiles on a 6-point Likert scale (very low, low, somewhat low, somewhat high, high, very high). Participants were instructed to rate the content of each scene tile based on how “informative or recognizable” it was. Participants were first given examples of two low-meaning and two high-meaning tiles, followed by a set of four practice trials to ensure understanding of the task. The practice trials contained two examples expected to score on the low-meaning side of the scale (1–3) and two examples expected to score on the high-meaning side of the scale (4–6). Seventy-nine participants were excluded based on the results of these diagnostic practice trials.

Each participant rated 40 tiles (20 of each spatial scale). Participants rated exactly one tile from each of the 40 scenes, and participants were prevented from completing the online experiment more than once. In total, the experiment took approximately 3 min and participants were compensated for completing the study.

Each tile was rated by three participants, and responses were averaged to produce a “meaning” rating for each tile. The average rating for each tile was then plotted at its center coordinate and smoothed using the variable-width gaussian filter. This process was completed at both spatial scales, and the average of these two maps was used as the scene’s final “meaning map”.

### Spherical sampling of equirectangular maps

To account for the distortion imposed by equirectangular map projections, which disproportionately represent scene regions at the poles, we sampled points uniformly on a sphere (N = 100,000; N = 77,050 after discarding locations not visible to passive viewers), projected those indices onto each equirectangular map (i.e., gaze, salience, meaning, and equator), and used the sampled values at these indices for analyses of spatial attention (Fig. 2)^47^. As a result, each individual location in an equirectangular map, per scene and condition, was treated as a separate observation (N = 6,164,000) in statistical analyses.

### Statistical analyses

To compute the relative contributions of visual salience and semantics in predicting gaze behavior, we built a linear mixed effects model using the lme4 package in R^48^. We included viewing condition (i.e., active vs. passive) and feature maps of scene content (i.e., salience, meaning, and equator map) as fixed effects and individual scenes as random effects. Specifically, for each scene, the model predicted the degree to which each feature map predicted the gaze map value at each location (each index from the N = 100,000 points uniformly sampled around the spherical image). Because gaze maps were generated at the group level^12,15^, individual subjects were not included as random effects in the model. Two-way interactions (i.e., salience by condition, meaning by condition) and the three-way interaction between salience, meaning, and condition were analyzed.

## Supplementary information

Supplementary Information
