# Supplementary material for: Active vision in immersive, 360° real-world environments
Source: Sci Rep. 2020 Aug 31;10:14304. doi: 10.1038/s41598-020-71125-4 (PMC7459302; doi:10.1038/s41598-020-71125-4)
Supplement: Supplementary file 1 — Supplementary Information [file 41598_2020_71125_MOESM1_ESM.pdf]

Active Vision in Immersive, 360° Real-World Environments

Amanda J. Haskins[1]*, Jeff Mentch[1,2], Thomas L. Botch[1], Caroline E. Robertson[1]

[1] *Department of Psychological and Brain Sciences, Dartmouth College, Hanover, NH 03755, USA*
[2] *McGovern Institute for Brain Research, Massachusetts Institute of Technology, Cambridge, MA 02139, USA*

Correspondence:

Address: 3 Maynard Street, Moore Hall, Hanover NH 03755
Phone: +1 (603) 646 9129
Email: ajh.gr@dartmouth.edu

Supplemental Information

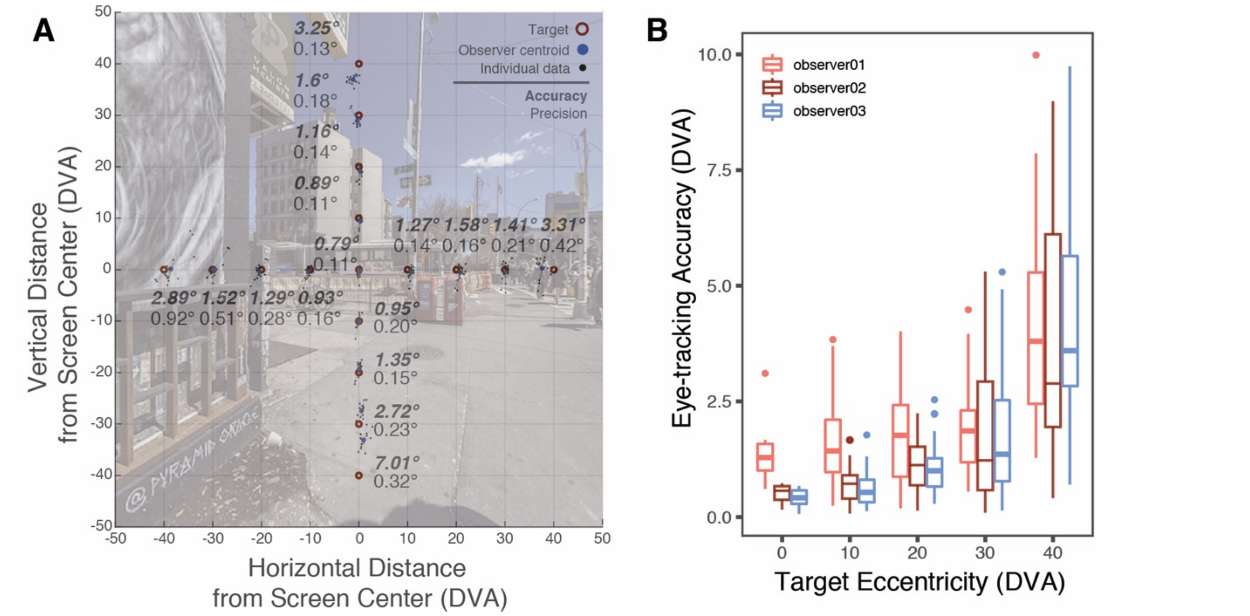

Supplemental Figure 1. Gaze measured in 360 is accurate and precise.
Three head-fixed participants made a series of saccades from a central fixation cross to 16 targets arranged in a cross and spanning the entire headmounted display field of view. Targets were located above, below, and to the right and left of screen center, and were located at distances of 10, 20, 30, and 40 DVA in each direction. Each observer completed this task six times, over separate sessions. The mean accuracy across observers and locations was 2.00 DVA +/- 0.38 STE, and the mean precision across observers and locations was 0.26 DVA +/- 0.05 STE. Eye-tracking accuracy decreased with eccentricity ($F(1, 6.126) = 58.175$, $p < 0.001$); however, gaze measured at screen center was accurate within <1 DVA, comparable to the reported accuracy of many mobile eye-tracking systems[1]. Image in Panel A is adapted from "Pyramid Oracle Panorama" by Nathan Tweti.

Supplemental Figure 2. Gaze map comparisons for active vs. passive viewing participants. Gaze maps were generated by plotting duration-weighted fixations for all participants in either the active viewing condition (left) or passive viewing condition (right). The dotted lines indicate regions that were not visible to passive viewers. These trimmed regions were not included in the analysis of participants' attention or in the comparison between passive saccade lengths and active viewing gaze shifts. Fixations made in these trimmed regions were included in analyses for which spatial location was irrelevant (i.e., the comparison between active and passive fixation durations and fixation numbers). All gaze comparisons can be viewed at https://bit.ly/2ZPxYxz. Adapted from original images by Nathan Tweti (nathantweti.com) and Samayou Kodomo (flickr.com/photos/samayoukodomo).

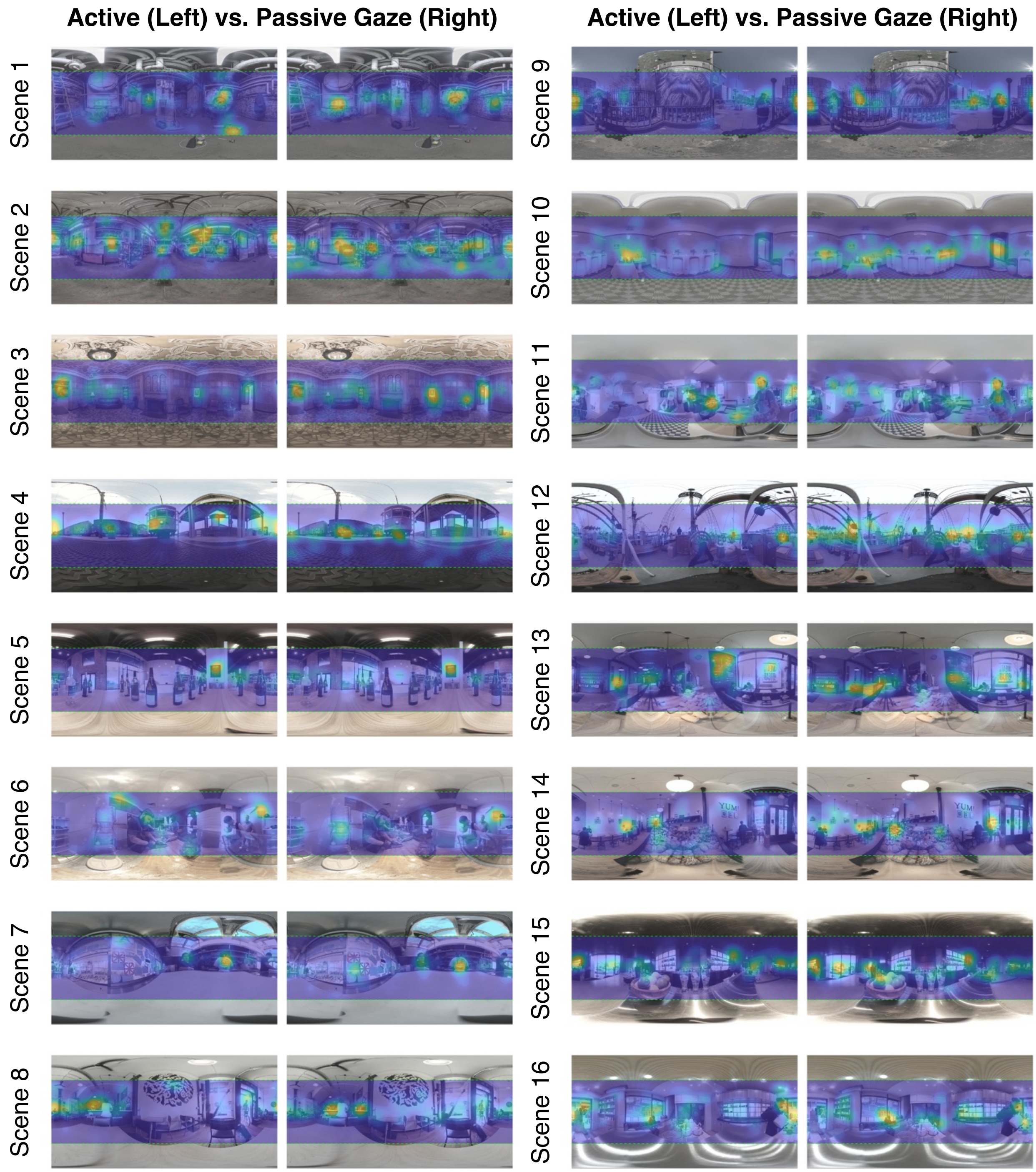

Supplemental Table 1: Control analysis (1/2) results summary. Meaning:salience:condition interaction remains significant when restricting analysis to the fields of view containing regions ranked in the top 50th percentile for meaning.

	NumDF	DenDF	*F* value	*p* value
condition	1	5935512	6.78E+03	< 0.001
meaning	1	5935478	6.89E+04	< 0.001
salience	1	5935534	1.98E+04	< 0.001
condition:meaning	1	5935512	2.21E+04	< 0.001
condition:salience	1	5935512	3.39E+00	0.0656
meaning:salience	1	5935548	6.07E+04	< 0.001
condition:equator	2	5935532	4.37E+05	< 0.001
condition:meaning:salience	1	5935512	4.34E+02	< 0.001

Supplemental Table 2: Control analysis (2/2) results summary. Meaning:salience:condition interaction remains significant when downscaling neighboring fixations made in the active condition to 5 seconds, the maximum duration a scene could be viewed passively.

	NumDF	DenDF	*F* value	*p* value
condition	1	6163960	1.46E+04	< 0.001
meaning	1	6163922	81998.17	< 0.001
salience	1	6163986	12431.52	< 0.001
condition:meaning	1	6163960	27103.56	< 0.001
condition:salience	1	6163960	375.89	< 0.001
meaning:salience	1	6163998	47519.71	< 0.001
condition:equator	2	6163980	470524.86	< 0.001
condition:meaning:salience	1	6163960	1.49E+03	< 0.001

Supplemental References

1. Cognolato, M., Atzori, M. & Müller, H. Head-mounted eye gaze tracking devices: An overview of modern devices and recent advances. *J. Rehabil. Assist. Technol. Eng.* **5**, 205566831877399 (2018).
